# Serological and Molecular Detection of Zoonotic Pathogens in European Bison (*Bison bonasus*) and Associated Ticks from Poland

**DOI:** 10.3390/pathogens15060562

**Published:** 2026-05-22

**Authors:** Anna Didkowska, Alejandro Navarro, Abel Dorrego, Jorge Martínez, Irene Martínez, Marta Kloch, Sergio González, Wanda Olech, Fatima Cruz-Lopez, Krzysztof Anusz, Nerea García

**Affiliations:** 1 Department of Food Hygiene and Public Health Protection, Institute of Veterinary Medicine, Warsaw University of Life Sciences (SGGW), 02-776 Warsaw, Poland; anna_didkowska@sggw.edu.pl (A.D.); krzysztof_anusz@sggw.edu.pl (K.A.); 2 VISAVET Health Surveillance Centre, Complutense University of Madrid, 28040 Madrid, Spain; angomez@ucm.es (A.N.); abeldorr@ucm.es (A.D.); jomart21@ucm.es (J.M.); irmartin@ucm.es (I.M.); goser@ucm.es (S.G.); ngarciab@ucm.es (N.G.); 3 Department of Animal Genetic and Conservation, Institute of Animal Sciences, Warsaw University of Life Sciences-SGGW, 02-786 Warsaw, Poland; marta_kloch@sggw.edu.pl (M.K.); wanda_olech@sggw.edu.pl (W.O.); 4 Department of Animal Health, Faculty of Veterinary Medicine, Complutense University of Madrid, 28040 Madrid, Spain

**Keywords:** *Anaplasma phagocytophilum*, *Brucella*, *Borrelia burgdorferi* sensu lato, hepatitis E virus, wildlife, European bison, tick

## Abstract

As wild ungulates, including European bison, increasingly share habitats with livestock, surveillance of infectious zoonotic agents in their populations is essential for both wildlife and public health. This study aimed to screen for selected zoonotic pathogens in European bison from Poland. Samples (blood, ticks, and spleen) were collected from 86 animals. Serum was used for serological testing using commercial ELISA kits for *Borrelia burgdorferi* sensu lato, *Brucella* spp., and hepatitis E virus (HEV); ticks were analysed by real-time PCR targeting *B. burgdorferi* s.l., *Anaplasma phagocytophilum*, and *Brucella* spp., and spleen samples from *Brucella*-seropositive animals were cultured. Serological analysis revealed that 53.9% of European bison were seropositive for *B. burgdorferi* s.l., while 25.3% showed seroreactivity against *Brucella* spp.; however, these findings were not supported by molecular or culture confirmation, suggesting possible non-specific reactions or past exposure. No serum samples were positive for HEV antibodies, and no *Brucella* spp. were isolated from spleen samples. Molecular analysis of ticks detected *B. burgdorferi* s.l. DNA in 4.8% of samples and sequencing confirmed *Borrelia garinii* in one case. In contrast, *A. phagocytophilum* DNA was detected in 59.0% of ticks. No ticks tested positive for *Brucella* DNA. These findings indicate substantial exposure of European bison to tick-borne pathogens, particularly *B. burgdorferi* s.l. and *A. phagocytophilum*. However, *Brucella* seropositivity should be interpreted with caution due to the lack of molecular or culture confirmation.

## 1. Introduction

Although the European bison (*Bison bonasus*) has successfully recovered from extinction and its population has increased substantially—resulting in its current Near Threatened (NT) classification [1,2]—the species remains vulnerable and continues to require sustained conservation efforts due to newly emerging threats [3]. As wild ungulates, including European bison, increasingly share habitats with livestock [4], surveillance of infectious agents circulating in European bison populations is essential for safeguarding both wildlife and public health within the One Health framework. Pathogens of particular concern include hepatitis E virus (HEV), *Borrelia burgdorferi* sensu lato, *Brucella* spp., and *Anaplasma phagocytophilum*, which are widespread in Europe and have previously been detected in various wild ruminant species.

Tick-borne bacterial pathogens such as *B. burgdorferi* s.l. and *A. phagocytophilum* are of major ecological and medical importance in Europe. *B. burgdorferi* s.l., the causative agent of Lyme borreliosis, is maintained in natural cycles involving ticks of the *Ixodes genus* and multiple mammalian hosts [5]. Similarly, *A. phagocytophilum* causes granulocytic anaplasmosis in both humans and animals and has been detected in a range of wild ruminants, which may serve as reservoir hosts [6]. The presence of these pathogens in European bison populations may reflect their exposure to infected tick populations circulating within forest ecosystems.

Brucellosis represents another important zoonotic disease affecting both domestic and wild animals, with major implications for animal health, public safety, and international trade [7]. *Brucella abortus* is the principal etiological agent of brucellosis in cattle, although it is also capable of infecting a wide range of wildlife species, including American bison [8]. Recent studies have reported seropositivity against *Brucella* spp. in more than one-third of tested European bison in Poland [9]. However, serological findings for *Brucella* spp. in wildlife should be interpreted cautiously due to the possibility of cross-reactivity with other Gram-negative bacteria, particularly when molecular or bacteriological confirmation is not available [10].

Hepatitis E is considered an emerging food-borne zoonotic disease in Europe caused mainly by HEV genotype 3 [11]. The main reservoirs are domestic pigs and wild boars, but also wild ruminants, such as deer have been associated with infections in humans [12]. Despite the detection of HEV or HEV-specific antibodies in domestic ruminants, there is currently no evidence supporting zoonotic transmission from these animals to humans, except for cases associated with camelids [13]. Regarding American bison, only a few studies have investigated HEV in this species, and available data indicate very low or negligible prevalence in these animals [12]. The most recent data on the lack of occurrence of HEV antibodies in European bison date back more than 10 years [14]. Given that hepatitis E is a significant public health concern worldwide, updated information on its prevalence is needed.

Given the aforementioned context, this study aimed to investigate the presence of HEV, *B. burgdorferi* s.l., *Brucella* spp., and *A. phagocytophilum* in European bison in Poland using both serological and molecular approaches. By assessing exposure to these pathogens, we aim to provide new, updated insights into the health status of this iconic species and its potential role in the epidemiology of selected zoonotic diseases. Understanding the circulation of these pathogens in European bison populations may contribute not only to wildlife conservation programs, but also to the surveillance of zoonotic risks at the wildlife–livestock–human interface.

## 2. Material and Methods

### 2.1. Sampling

**Figure 1:**
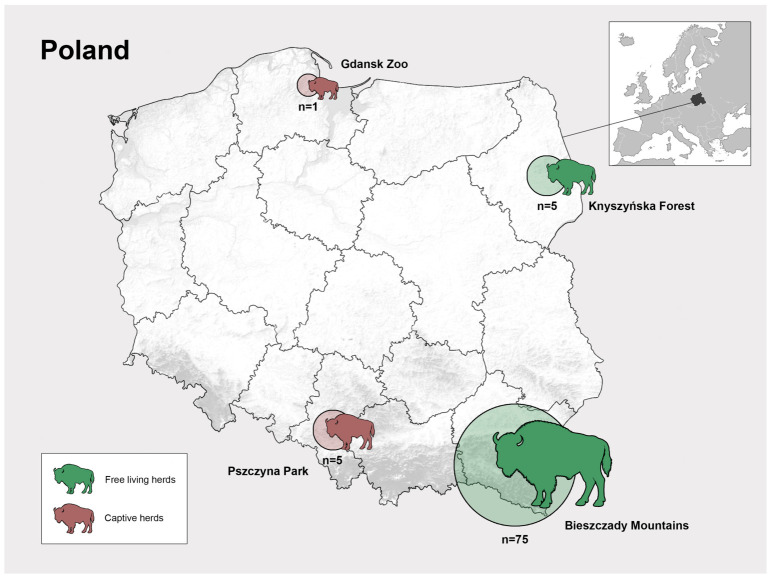
Geographic distribution of sampled European bison in Poland. Locations of sampled animals, including free-ranging populations from the Bieszczady Mountains and Knyszyńska Forest, and captive populations from Pszczyna and Gdańsk Zoo.

Samples were collected from 86 European bison, 48 males and 38 females, ranging in age from 7 days to 24 years old (mean age: 10.7 years), between 2021 and 2025. Age was determined based on the body mass, and appearance of teeth and horns [15]. No animals were sacrificed for the purpose of this study. Animals originated from free-living herds in the Bieszczady Mountains (*n* = 75) and the Knyszyńska Forest (*n* = 5), as well as from captive herds in Pszczyna (*n* = 5) and Gdańsk Zoo (*n* = 1), represented in Figure 1.

A total of 63 serum samples, 80 spleen samples, and 105 ticks (collected from 27 animals) were obtained from culled or dead animals. No animals were sacrificed specifically for this study. Samples were collected from animals found dead or culled during official population management activities. According to local regulations, the use of these post-mortem samples did not require a specific permit for scientific purposes, as previously described [16]. Differences in sample numbers were related to sample availability and preservation status from each animal.

Blood samples were collected from the jugular vein, heart, or body cavities, while ticks and spleen fragments were collected during routine post-mortem veterinary examinations. All samples were transported to the laboratory under refrigerated conditions, and ticks and spleen samples were subsequently frozen at −20 °C. Blood tubes were centrifuged; serum was separated and stored at −20 °C. Samples were then transported in dry ice to the VISAVET Health Surveillance Centre, where they were processed in a biosafety level 3 (BSL-3) facility.

Serum samples were used for serological tests; ticks were used for molecular results and spleen samples were used for *Brucella* spp. testing.

### 2.2. Serology

Serum samples were thawed at room temperature and analyzed using a commercial indirect enzyme-linked immunosorbent assay (ELISA) kits designed to detect specific antibodies against *B. burgdorferi* s.l. (ID Screen Borreliosis double antigen multispecies, Innovative-diagnostics, Grables, France), *Brucella* spp. (Ingezim *Brucella* Compac 2.0, Madrid, Spain), and HEV (ID Screen Hepatitis E Indirect multiespecies, Innovative-diagnostics, Grables, France) according to the manufacturer instructions. Positive and negative controls supplied with the commercial kits were included in each run. The cut-off values for determining seropositivity were applied in accordance with the manufacturer’s recommendations for bovine samples, due to the close phylogenetic relationship between European bison and domestic cattle. However, these assays have not been specifically validated for European bison, and results should therefore be interpreted cautiously.

### 2.3. DNA Extraction and Molecular Screening for Brucella *spp.*, B. burgdorferi *s.l.* and A. phagocytophilum

Ticks collected from 27 European bison were included in the study. The number of ticks analysed per animal varied from one to 12. In total, 105 ticks were processed. Prior to the extraction, they were cut as small as possible, and their exoskeleton was crushed. Samples were homogenised in 180 µL of ATL buffer, and DNA was extracted manually using a commercial extraction kit, the DNeasy Blood and tissue Kit (Qiagen, Hilden, Germany), following the manufacturer’s instructions.

The extracted DNA was used for molecular screening. Detection of all targets was performed using previously described real-time PCR assays for *Brucella* spp. [17], *B. burgdorferi* s.l. [18], and *A. phagocytophilum* [19].

### 2.4. Sequencing

The characterization of the five positive samples for the *B. burgdorferi* s.l. complex was performed through the amplification and subsequent sequencing of the 5S-23S ribosomal RNA intergenic spacer, following the PCR protocol described by [18]. The resulting PCR products were sequenced by SECUGEN S.L. (Madrid, Spain) using capillary electrophoresis (Sanger method). For species identification, the obtained sequences were compared against the NCBI nucleotide database using the BLASTn tool (National Center for Biotechnology Information, Bethesda, MD, USA; accessed on 13 February 2026).

### 2.5. Culture (Brucella)

Spleen tissue samples obtained from animals that yielded positive ELISA results were subjected to microbiological culture. Frozen spleen samples were thawed, and approximately 1.5 cm^2^ of tissue was excised and finely minced. The samples were then placed into stomacher bags with filters and combined with 10 mL of phosphate-buffered saline (PBS). Homogenization was performed mechanically in a stomacher for 10 min. Following homogenization, a 0.1 mL aliquot of the homogenate was inoculated onto two selective media—Farrell [20] and CITA [21]—prewarmed prior to use. Plates were incubated at 37 °C under a CO_2_ atmosphere.

Given the expected low bacterial load, an enrichment step in liquid culture medium was performed in addition to direct culture, following the recommendations of the WOAH Manual 2023, Brucellosis, Chapter 3.1.4. [22]. The procedure consisted of mixing 1 mL of the original homogenate (PBS + tissue) with 10 mL of enrichment medium. Liquid cultures were incubated under a CO_2_ atmosphere for a total of six weeks, with weekly subcultures performed on both Farrell and CITA media under identical incubation conditions.

After cultivation, DNA was extracted from the homogenized spleens following the extraction and PCR process as used in ticks for *Brucella* spp.

### 2.6. Statistical Analysis

Descriptive statistical analyses were performed. Apparent seroprevalence was calculated as the proportion of seropositive animals among those tested. Ninety-five percent confidence intervals (95% CI) were calculated using Sterne’s exact method [23].

## 3. Results

Serological analysis revealed high seropositivity for *B. burgdorferi* s.l. and lower seroreactivity against *Brucella* spp., whereas no samples were positive for HEV antibodies (Table 1). Overall, most analyzed animals were positive in at least one assay, with nine animals testing positive for both *B. burgdorferi* s.l. and *Brucella* ELISAs.

Molecular analysis of ticks collected from European bison detected both *B. burgdorferi* s.l. and *A. phagocytophilum* DNA, whereas no ticks tested positive for *Brucella* spp. (Table 2). Sequencing and bioinformatic analysis of a single *Borrelia*-positive tick sample with sufficient quality showed 100% sequence identity over 21% query coverage (E-value: 0.013) with several *Borrelia garinii* strains deposited in GenBank, including isolates RUS/Nov15-3052Drt (Accession: KX759017.1) and TIGMIC-005 (Accession: PP746237.1).

All Borrelia-positive samples showed concurrent positivity for *A. phagocytophilum*. At least one tick positive for *A. phagocytophilum* was detected in 20 of the 27 animals tested, and positivity for at least one analyzed target was common among tested ticks (Table 2).

## 4. Discussion

Our results indicate a high exposure of European bison to zoonotic tick-borne pathogens, particularly *A. phagocytophilum* and *B. burgdorferi* s.l., suggesting active circulation of these agents in the environment. The detection of antibodies against *Brucella* spp. in the absence of molecular or culture confirmation should be interpreted cautiously and does not allow definitive conclusions regarding active infection in the studied populations. Conversely, the absence of serological evidence of HEV infection indicates that this pathogen likely plays a minor or negligible role in the studied populations. Taken together, these findings suggest that European bison may serve as useful indicators of tick-borne pathogen circulation in forest ecosystems.

Our study revealed a molecular prevalence of *A. phagocytophilum* in ticks (59.0%) comparable to that reported in previous investigations, while representing one of the largest sample sizes analyzed to date. Earlier studies on ticks collected from European bison reported prevalence ranging from 15.4% to 67.0% [24,25,26,27,28,29]. In contrast, studies based on European bison tissues have generally reported lower prevalences (26.0–40.0%) [28,30,31]. Notably, in the present study, at least one tick collected from each examined European bison tested positive for *A. phagocytophilum*, indicating widespread exposure to infected vectors. Overall, these findings support the continued circulation of *A. phagocytophilum* in European bison habitats in Poland, although temporal trends cannot be inferred from the present study.

To our knowledge, this study represents the first comprehensive investigation of *B. burgdorferi* s.l. exposure in European bison combining serological and molecular data. The high seroprevalence observed (53.9%), together with the detection of *Borrelia* DNA in ticks (4.8%), suggest widespread exposure of European bison to spirochetes circulating in their environment. However, it is likely that other wildlife species act as the primary reservoirs of *B. burgdorferi* s.l., while European bison primarily serve as hosts supporting tick populations rather than as key amplifying hosts [32]. This interpretation is supported by reports from Poland documenting *Borrelia* spp. in wildlife and ectoparasites, including carnivores (8.8–23.5%) [33,34], deer keds (14.0%) [35], and ticks (23.0–26.1%) [36,37]. In contrast, the prevalence of *Borrelia* spp. in ticks collected from wild cervids has been reported to be lower (3.3%) and closer to the values observed in our study [38]. High seroprevalence is also consistent with reports from other wild ruminant species in Europe [39,40].

The discrepancy between the high seropositivity observed in European bison and the relatively low detection rate of *Borrelia* DNA in ticks may be explained by several factors. Antibodies against *B. burgdorferi* s.l. may persist for prolonged periods after exposure, meaning that serological positivity can reflect previous contact with the pathogen rather than current infection or recent exposure [41,42]. In contrast, PCR detection in ticks reflects only the presence of pathogen DNA at the specific time and location of sampling. Therefore, temporal mismatches between past exposure and current vector infection rates are likely. In addition, ecological and seasonal variation in tick abundance and pathogen prevalence, as well as differences among tick developmental stages and feeding status, may influence molecular detection rates [41,42]. In addition, methodological limitations of serological testing should also be considered. Regarding serology, ELISAs remain the primary screening tools for the detection of antibodies against *B. burgdorferi* s.l. [43,44]. ELISAs based on whole-cell lysate antigens are known to produce false-positive results due to cross-reactivity and may require confirmation by Western blotting. In contrast, the ELISA used in the present study employs recombinant antigens, which may provide improved specificity compared to whole-cell lysate–based assays [43,44]. Therefore, confirmatory testing using methods such as Western blotting would be useful to further validate the serological findings obtained in this study.

Sequencing of one *Borrelia*-positive tick confirmed the presence of *B. garinii*, a genospecies commonly associated with avian hosts [45,46], highlighting the ecological complexity of local transmission cycles. The presence of *B. garinii* at low prevalence is consistent with previous findings from Poland [47].

In contrast, the seroprevalence of antibodies against *Brucella* spp. (25.3%) was not supported by molecular detection or bacterial isolation. All tested tissue samples were negative by PCR and culture, and no *Brucella* DNA was detected in ticks or tissues. However, negative PCR and culture results do not definitively exclude infection, particularly in wildlife species, where bacterial burden may be low, tissue distribution heterogeneous, and culture sensitivity limited [48]. The lack of concordance between serological and direct detection methods may also result from cross-reactivity, particularly with antibodies elicited by *Yersinia enterocolitica* O:9, *Escherichia coli* O:157, *Stenotrophomonas maltophilia*, and *Salmonella urbana*, or non-specific reactions [49,50]. The seroprevalence observed in this study was lower than that reported previously in European bison in Poland (36.0%) [9]. From both conservation and public health perspectives, the lack of molecular and bacteriological confirmation suggests that there is currently no clear evidence of widespread active *Brucella* infection in the studied populations; however, continued surveillance remains advisable.

No European bison tested positive for antibodies against HEV, in agreement with earlier studies conducted more than a decade ago [14]. This finding is not unexpected, as HEV is well documented in wild boar populations in Poland [51,52], whereas HEV infection in wild ruminants appears to be sporadic [53,54,55,56]. To date, only limited studies have reported seropositivity for HEV in captive wild ruminants, with buffaloes in India and Egypt showing prevalence rates of 100% and 14%, respectively, and American bison (*Bison bison)* in the United States exhibiting a seroprevalence of 4.6% [57,58,59].

This study has some limitations. The limited and uneven sampling distribution, including the restricted geographic origin of most animals and the partial availability of ticks, may affect the generalizability of the results. In addition, serological cross-reactivity, particularly in the case of *Brucella* spp., cannot be completely excluded. The commercial serological assays used in this study were validated for domestic species but not specifically for European bison, which should be considered when interpreting seroprevalence estimates. Future studies including larger and more standardized sample sizes, longitudinal monitoring, and molecular characterization directly from European bison tissues would help to better clarify the epidemiological role of this species in the maintenance and circulation of zoonotic pathogens. Finally, molecular characterization was only possible for one *Borrelia*-positive sample, limiting further assessment of Borrelia diversity in the studied population.

Despite these limitations, the present study provides valuable updated information for health monitoring and disease surveillance in European bison populations in Poland. Our findings highlight the complex interactions between European bison, tick vectors, and zoonotic pathogens within forest ecosystems, reinforcing the importance of wildlife surveillance within the One Health framework. The combined use of serological, molecular, and cultural methods underscores that antibody prevalence does not necessarily reflect current infection and emphasizes the importance of integrating complementary diagnostic approaches in wildlife disease studies. These data may contribute to future epidemiological surveillance programs and risk assessment strategies at the wildlife–livestock–human interface.

## Figures and Tables

**Table 1:** Seroprevalence of selected zoonotic pathogens in European bison from Poland. Number and percentage of seropositive animals for *Borrelia burgdorferi* sensu lato, *Brucella* spp., and hepatitis E virus (HEV), based on ELISA results. Ninety-five percent confidence intervals (95% CI) were calculated using Sterne’s exact method. Total indicates animals positive for at least one pathogen.

ELISA Target	Total Samples	Positive Serum Samples (n, (%))	CI (95%)
*Brucella* spp.	63	16 (25.3)	15.6–38.0
HEV		0 (0.0)	-
*B. burgdorferi* s.l.		34 (53.9)	41.2–65.9
**Total**	**63**	**50 (79.3)**	**67.8–87.5**

**Table 2:** Molecular detection of zoonotic pathogens in ticks collected from European bison. Number and percentage of PCR-positive tick samples for *Borrelia burgdorferi* sensu lato, *Anaplasma phagocytophilum*, and *Brucella* spp. Ninety-five percent confidence intervals (95% CI) were calculated using Sterne’s exact method. Total indicates ticks positive for at least one pathogen.

PCR Target	Total Ticks	Positive Tick Samples (n, (%))	CI (95%)
*Brucella* spp.	105	0 (0.0)	-
*A. phagocytophilum*		62 (59.0)	49.0–68.1
*B. burgdorferi* s.l.		5 (4.8)	1.90–10.8
**Total**	**105**	**67 (63.8)**	**54.3–72.4**

Additionally, *Brucella* spp. were not isolated by culture (0/16; 0%) nor detected by PCR in any of the homogenized spleen samples.

## Data Availability

All relevant data are included in the manuscript. Additional supporting data, including raw sequence chromatograms, are available from the corresponding authors upon reasonable request.
